# Supplementary material for: Metal Ion-Chelated Tannic Acid Coating for Hemostatic Dressing
Source: Materials (Basel). 2019 Jun 3;12(11):1803. doi: 10.3390/ma12111803 (PMC6600752; doi:10.3390/ma12111803)
Supplement: Supplementary file 1 [file materials-12-01803-s001.pdf]

Supporting information for

Metal Ion-Chelated Tannic Acid Coating for Hemostatic Dressing

Bing Song [1], Liwei Yang [1], Lulu Han [1,*] and Lingyun Jia [1]

[1] Liaoning Key Laboratory of Molecular Recognition and Imaging, School of Bioengineering, Dalian University of Technology, Dalian 116023, PR China; songbing@mail.dlut.edu.cn(B. S.); yanglw@mail.dlut.edu.cn(L. Y.); luluhan@dlut.edu.cn (L. H.); lyjia@dlut.edu.cn(L. J.)

* Correspondence: luluhan@dlut.edu.cn; Tel.: +86-411-84706316

Received: date; Accepted: date; Published: date

Table of Contents

Table S1. Physicochemical properties of the three model proteins and their coupling efficiency with FITC.. 4

Figure S1. Absorption spectrum of three FITC labeled proteins. 5

Figure S2. Fluorescence intensity of model protein adsorption on the TA coated-silicon slide. 6

Preparation of Fluorescently Labeled Proteins

Labeling of bovine serum albumin (BSA), human Immunoglobulin G (IgG) and fibrinogen (Fgn) with fluorescein isothiocyanate (FITC) was performed according to the instruction of the FITC manufacturer. Briefly, FITC was dissolved in anhydrous DMSO at 1 mg mL^{-1}, and a solution of 2 mg mL^{-1} of protein in 0.1 M sodium carbonate buffer, pH 9.8 was prepared. For each 1 mL of protein solution, 50 μL of FITC solution was added slowly, while gently and continuously stirring the protein solution. The reaction was incubated in the dark for 12 h at 4 ºC. Afterwards, excess FITC was removed by dialysis membrane (7 kDa).

The ratio of fluorescein to protein (F/P) of the product was estimated by measuring the absorbance at 498 nm and 280 nm via a UV spectrophotometer (Figure S1). The F/P molar ratio is defined as the ratio of moles of FITC to moles of protein in the conjugate. It was calculated from the following formulas:

$$MolarF/P = \frac{MW}{389} \times \frac{A_{498}E^{0.1\%}}{195[\mathrm{A}_{280} - (0.35 \times A_{498})]}$$

Where, MW is molecular weight of the protein; $E^{0.1\%}$ is the absorption at 280 nm of a protein at 1.0 mg mL^{-1}; A_{280} and A_{498} is the absorbance of the conjugate sample at 280 nm and 498 nm, respectively.

Table S1. Physicochemical properties of the three model proteins and their coupling efficiency with FITC. MW: molecular weight; pI: isoelectric point; F/P molar ratio: the ratio of mole of FITC to mole of protein in the conjugate.

Protein	BSA	IgG	Fgn
**MW(kDa)**	66	150	340
**pI**	4.6	8.6	5.6
**F/P molar ratio**	3	3	2
**Shape**	Prism shape	Sphere	Cylinder
**Dimensions (nm)**	4.0*4.0*14.0	6.6*7.7*10.1	diameter 6.5, length 47.5
**Plasma concentration (mg/mL)**	40	6.5-16.5	2.0-4.0
**PDB ID**	HSA : 1E78<br>Not find yet BSA	1IGT	3GHG
	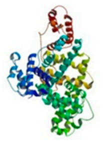	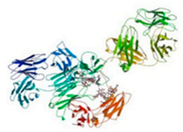	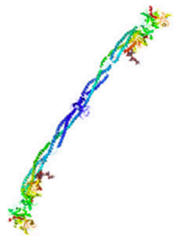

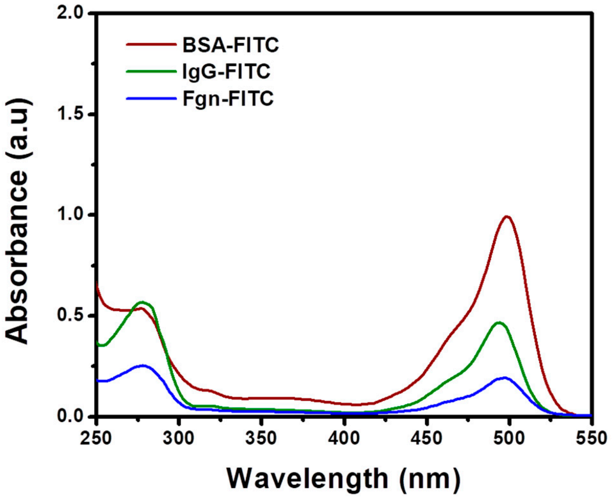

Figure S1. Absorption spectrum of three FITC labeled proteins.

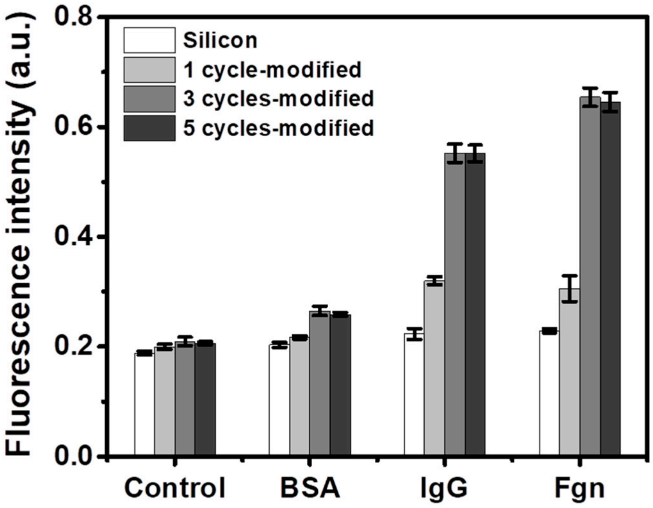

Figure S2. Fluorescence intensity of model protein adsorption on the TA coated-silicon slide.
